# Early detection of ICU-acquired weakness in septic shock patients ventilated longer than 72 h

**DOI:** 10.1186/s12890-022-02193-7

**Published:** 2022-12-06

**Authors:** Caroline Attwell, Laurent Sauterel, Jane Jöhr, Lise Piquilloud, Thierry Kuntzer, Karin Diserens

**Affiliations:** 1 Acute Neuro-Rehabilitation Unit, Lausanne University Hospital, Lausanne, Switzerland; 2 Lausanne University Hospital, Lausanne, Switzerland; 3 Adult Intensive Care Unit, Lausanne University Hospital and University of Lausanne, Lausanne, Switzerland; 4 Nerve-Muscle Unit, Neurology Service, Lausanne University Hospital, Lausanne, Switzerland

**Keywords:** Critical illness polyneuropathy, Critical illness myopathy, Sepsis, Ventilation weaning failure, Early mobilization, Peroneal-nerve test, PENT, CMAP

## Abstract

**Purpose:**

*ICU-acquired weakness*, comprising Critical Illness Polyneuropathy (CIP) and Myopathy (CIM) is associated with immobilization and prolonged mechanical ventilation. This study aims to assess feasibility of early detection of CIP and CIM by peroneal nerve test (PENT) and sensory sural nerve action potential (SNAP) screening in patients with septic shock and invasively ventilated for more than 72 h.

**Methods:**

We performed repetitive PENT screening from 72 h after intubation until detecting a pathological response. We tested SNAPs in pathological PENT to differentiate CIP from CIM. We performed muscle strength examination in awake patients and recorded time from intubation to first in-bed and out-of-bed mobilization.

**Results:**

Eighteen patients were screened with PENT and 88.9% had abnormal responses. Mean time between intubation and first screening was 94.38 (± 22.41) hours. Seven patients (38.9%) had CIP, two (11.1%) had CIM, one (5.6%) had CIP and CIM, six (33.3%) had a pathological response on PENT associated with ICU-acquired weakness (but no SNAP could be performed to differentiate between CIP and CIM) and two patients had (11.1%) had no peripheral deficit. In patients where it could be performed, muscle strength testing concorded with electrophysiological findings. Twelve patients (66.7%) had out-of-bed mobilization 10.8 (± 7.4) days after admission.

**Conclusion:**

CIP and CIM are frequent in septic shock patients and can be detected before becoming symptomatic with simple bedside tools. Early detection of CIP and CIM opens new possibilities for their timely management through preventive measures such as passive and active mobilization.

**Supplementary Information:**

The online version contains supplementary material available at 10.1186/s12890-022-02193-7.

## Introduction

Critical illness polyneuropathy (CIP) and critical illness myopathy (CIM) are part of a clinical entity called *intensive care unit* (*ICU) acquired weakness (ICUAW)*, a frequent complication of critical illnesses [1] shown to delay ventilator weaning and discharge from the ICU, prolong the rehabilitation period and general hospitalization stay and increase risk of death [2–4]. Identified risk factors for CIP and CIM are multiple organ failure (MOF), sepsis and prolonged mechanical ventilation. Incidence of ICU-acquired weakness in patients with severe sepsis was shown to be significantly higher than in other patient groups (64% sepsis vs 30% other) [5]. In addition, ICU-acquired weakness was diagnosed in up to 67% of patients with long-term ventilation and seemed to be the cause as well as a consequence of prolonged mechanical ventilation [3]. Indeed, respiratory muscle weakness due to CIP or CIM impaired weaning from mechanical ventilation [6, 7] while risk of developing ICU-acquired weakness increased with the duration of exposure to mechanical ventilation [1, 5, 8].

An early identification of persons at risk for ICU-acquired weakness would allow timely preventive interventions, such as treating conditions leading to multi organ failure, avoiding unnecessary deep sedation, controlling excessive blood glucose levels and corticosteroid administration and promoting early mobilization as preventive strategy [9–11]. In clinical practice, ICU-acquired weakness is usually diagnosed by muscle strength testing that can only be performed on awake and cooperative patients. Although this tool is necessary to confirm diagnosis, its use for early screening is limited by the need to await patient awakening, especially in subjects remaining in a critical state for days to weeks; thereby, delaying any possible targeted preventive measures to avoid development of ICU-acquired weakness. Electrophysiological studies, on the other hand have the advantage that they do not require patient cooperation and can therefore be pursued systematically on at-risk patients in the early phase of hospitalization [12, 13].

However, complete electrophysiological evaluation is too fastidious for screening critically ill patients [12, 13]. Latronico et al. proposed therefore, a simplified screening method using motor peroneal nerve test (PENT) to identify motor conduction loss [13], allowing early testing on at-risk populations. PENT is a screening method allowing diagnosis of CIP and CIM with a sensitivity of 100% and specificity of 85.2% [13]. It consists of measuring the compound muscle action potential (CMAP) of the common peroneal nerve. Stimulation occurs through an active electrode placed on the belly of the extensor digitorum brevis muscle, and the recording electrode is placed on its distal tendon. In the presence of polyneuropathy or myopathy, the CMAP amplitude is reduced. To differentiate between CIP and CIM, other features must be assessed. For example, a sensory nerve action potential (SNAP) can be recorded at the sural or median nerve and a decreased amplitude of both CMAP and SNAP indicate a polyneuropathy as they testify to injury of both motor and sensory nerves. On the other hand, a delayed muscle contraction with a normal SNAP suggests myopathy.

Earlier identification of ICU-acquired weakness through neurophysiological testing allows gaining several days during which targeted preventive methods could be applied before its obvious clinical manifestation. It would thereby open new perspectives for earlier management of CIP and CIM through preventive measures, like intense passive and active early mobilization. Indeed, early mobilization methods are constantly progressing through the introduction and improvement of robotic devices such as cycloergomatry, MOTOmed-letto® and Erigo®, for example facilitating coma patient rehabilitation by moving the patient into an upright position [14, 15], thereby improving muscle force, endogenous catecholamine production and prevention of complications of bed rest [14–17]. However, mobilization practices seem to be generally lacking, especially in the category of ventilated patients, although they could improve their prognosis [9].

The aim of this study is to assess the feasibility of the simplified screening method of Latronico et al. for early detection of CIP and CIM in a population known to be at high risk of developing these two conditions: patients admitted to the ICU with septic shock and mechanically ventilated for more than 72 h. This research also aims to describe the current mobilization practices on patients who develop CIP and CIM during their acute ICU stay.

## Methodology

### Study design, setting and participants

This study took place in the adult ICU of the Lausanne university hospital. We recruited patients over a six-month period from January 1 to June 30, 2019. Patients admitted to the ICU who either had septic shock or who developed septic shock during ICU stay and who had invasive ventilation for ≥ 72 h were eligible for the study. Inclusion took place during the period of mechanical ventilation, patients could therefore only be included if they presented septic shock while being mechanically ventilated. We defined septic shock according to the Surviving Sepsis Campaign guidelines 2017 [18]. Exclusion criteria were pregnancy, age below 18 years and patient with previously known muscle or nerve disorders of central or peripheral origin (such as stroke, neurodegenerative diseases, peripheral neuropathy or muscle dystrophy) to eliminate potential confounding conditions. We also excluded patients with lower-limb disorders precluding nerve and muscle conduction studies, such as massive edema, fractures, lower-limb amputation and lower-limb immobilization from a plaster. In addition, we excluded patients hospitalized for more than 14 days before intubation, patients with a known functional disability of four or more on the Modified Rankin Scale [19] and patients admitted to the ICU for severe burns.

### Ethics statement

The clinical study protocol n° 2018–01,233 was approved by the local ethics committee, Commission cantonale d'éthique de la recherche sur l'être humain – CER-VD. As soon as a patient matched the inclusion criteria, the clinician in charge of the patient authorized inclusion following the procedure for inclusion in an emergency. We obtained written informed consent from all surviving patients as soon as they had capacity, or from the relatives or legal representatives of deceased patients. We conducted clinical investigations according to the principles expressed in the Declaration of Helsinki.

### Data collection, variables and clinical diagnosis

**Fig. 1:**
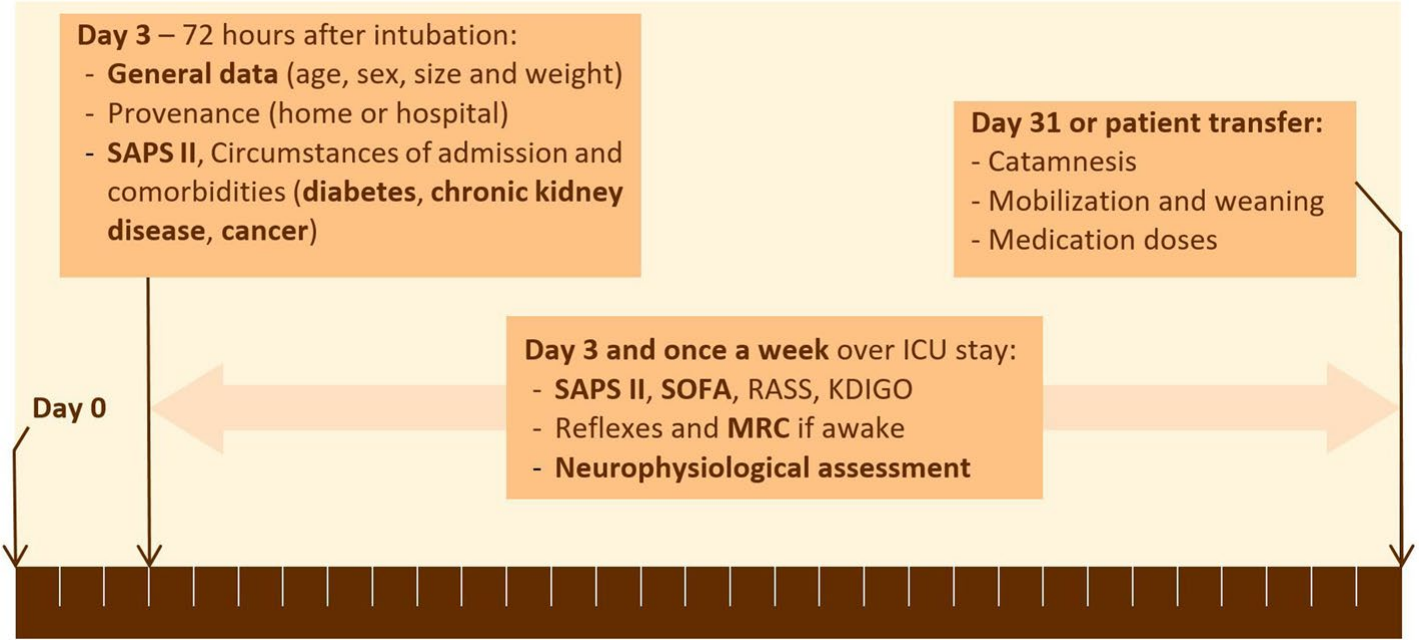
Course of investigation and measures during the ICU stay

We evaluated included patients on the day following 72 h of intubation (day 3, Fig. 1). We then examined patients once a week according to the investigation plan (Fig. 1). All clinical scores were calculated with the values collected from the clinical database of our ICU. We used the following clinical scores: Simplified Acute Physiology Score II (SAPS II) [20] to monitor illness severity at ICU admission and predict patient outcome, Sepsis-Related Organ Failure Assessment (SOFA) [21] to grade severity of organ failure, Kidney Disease Improving Global Outcomes (KDIGO) [22] to grade acute kidney injury and Richmond agitation-sedation scale (RASS) [23] to grade state of sedation.

For each patient, we noted the cumulated doses of opiates, propofol and midazolam and recorded mobilization onset and schedule from the ICU database, focusing on time between intubation and first passive and active mobilizations in-bed, sitting in the bed or out-of-bed. Passive in-bed mobilization consisted of two methods, a passive mobilization of the patient’s articulations by a physiotherapist in a daily 15-min session and a passive mechanical mobilization with the Motomed® cyclo-ergometer.

We assessed factors associated with CIP and CIM, such as length of mechanical ventilation and extubation failure [6–8, 24, 25]. We considered extubation failure as any case of endotracheal tube removal followed by re-intubation within 72 h. As ICU-acquired weakness is associated with increased weaning failure due to respiratory muscle weakness [6, 7], we assessed weaning difficulty in our patient cohort using the score proposed by the WIND study. This classification of weaning situations offers a new insight into patient outcomes, with a poorer prognosis correlating with a longer ventilation duration. The WIND study proposes the following categories: WIND 0 – no weaning: no extubation or extubation attempt, WIND 1 – short weaning: successful weaning process in less than one day after the first weaning attempt, WIND 2 – difficult weaning: weaning within one day to one week after the first weaning attempt and WIND 3 – prolonged weaning: weaning taking more than one week after the first weaning attempt [25]. We also recorded length of stay in the ICU, patient destination after the ICU and re-admission rate to the ICU.

Patients underwent neurological examination at inclusion (day 3), then weekly and finally, on their last day in the ICU. We assessed reflexes (brachial biceps, quadriceps, and Babinski’s sign) before electrophysiological examination. We tested muscle strength of three muscle groups of the four limbs using the Medical Research Council (MRC) scale (from 0 to 5) with a cutoff value defining muscle weakness below 48/60 points [26, 27]. According to the criteria proposed by Bolton and Latronico [28], clinical diagnosis of ICU-acquired weakness is established in the presence of critical illness with multi-organ failure and either limb weakness or difficult ventilator weaning, neither explained by heart or lung disease. In our study, limb weakness was concluded with an MRC score below 48, and difficult ventilator weaning with a WIND score of 2 (difficult weaning) or 3 (prolonged weaning) without an explanatory heart or lung condition. The clinical investigator was not the physician in charge of the therapeutic decisions.

### Neurophysiological diagnosis

We recorded motor nerve conduction using peroneal nerve test (PENT) [13], which we performed on the day of inclusion (day 3) or as soon as the patient’s condition allowed performing a test without artefacts caused by agitation or severe edema hindering the nerve conduction study. Patients under muscle-blocking drugs were tested only after their complete interruption and assessment of a positive train-of-four muscle response [29].

CA, a technician trained in neurophysiology and with sufficient clinical experience performed PENT screening. Other nerve conduction studies were performed and interpreted by a board-certified physician in neurophysiology (TK) with extensive clinical experience who validated and interpreted all neurophysiological tests of each patient. We performed tests on the patient lying in bed. Sources of artefacts such as support surfaces preventing decubitus ulcer were shortly deactivated for the procedure. The patient’s skin was wetted with water to ensure optimal contact of the active electrode.

PENT measures compound muscle action potentials (CMAPs) with surface electrodes after electrical stimulation of the peroneal nerve at the patient’s ankle. We placed the active electrode on the belly of the extensor digitorum brevis muscle and the indifferent electrode on the distal tendon of the recorded muscle. The stimulus intensity was gradually increased until we obtained the maximal CMAP. The CMAP negative peak amplitude was measured, and an abnormal amplitude was defined as < 2.5 mV. When the test showed the normal response (> 2.5 mV), we repeated it on the contralateral limb. Amplitudes > 2.5 mV on both sides defined a normal condition. We considered an unpaired response on one side an abnormal condition, indicating further testing.

Further testing involved wider investigation in a second session where we measured other CMAPs (distal stimulation of the median nerve at the wrist to the abductor pollicis brevis muscle and stimulation of the musculocutaneous nerve at the arm to biceps brachialis muscle). In addition, sensory nerve action potentials (SNAPs) following stimulation of the sural nerve at the ankle [5] (stimulation to the lower third of the leg with SNAP recording bilaterally on the lateral malleolus with surface electrodes). The normal sural SNAP had peak-to-peak amplitude ≥ 5 μV.

We considered CIP diagnosis when CMAPs and SNAPs were of low amplitude. We defined CIM when CMAPs were of low amplitude together with an increased duration, with a normal sural SNAP [30, 31].

### Statistical methods and follow-up

We did a purely descriptive statistical analysis to summarize our data and results are reported as mean (SD) or median (IQR) depending on the data distribution assessed visually using histograms. We used numbers and percentages to describe proportions. We used the program Excel (version 2016) for statistical analysis.

## Results

### Patient screening and demographics

**Fig. 2:**
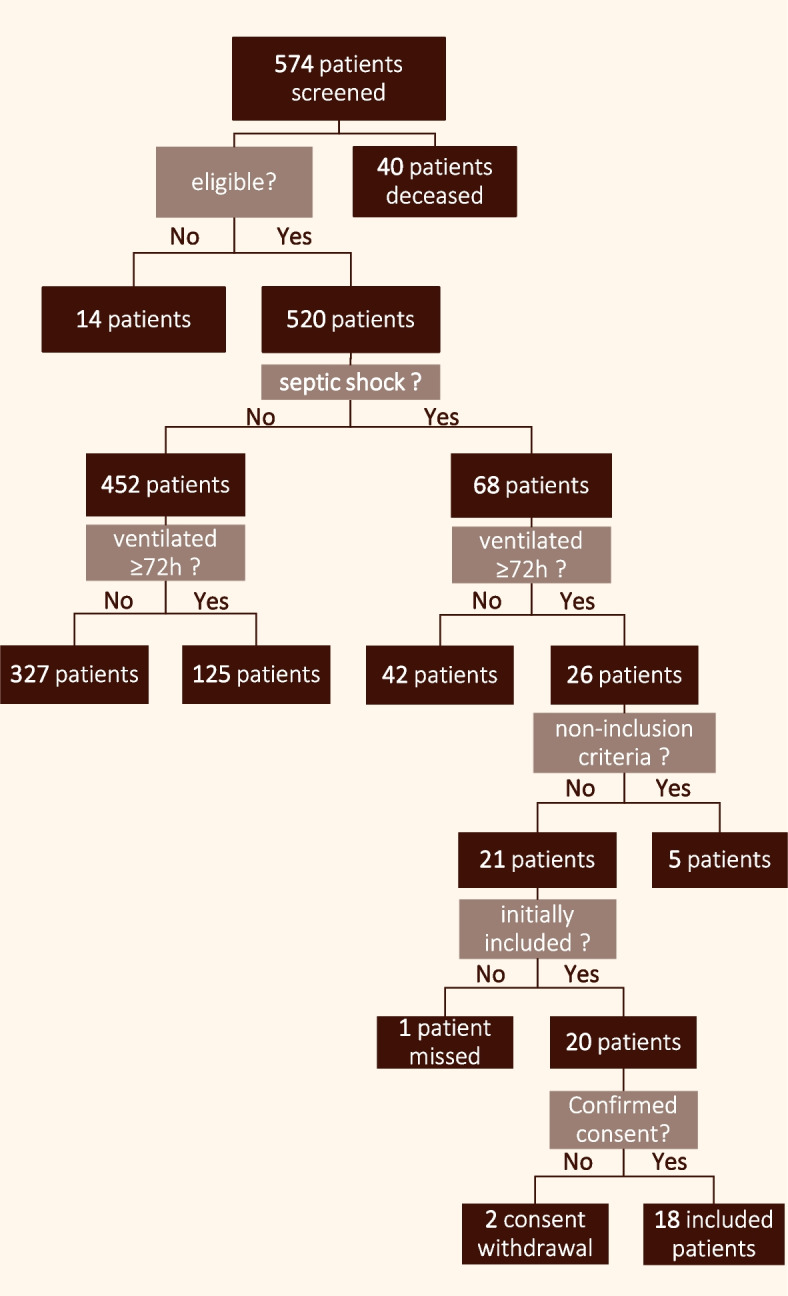
Flow diagram of patient screening and inclusion

Over the six months of investigation, we screened 574 potentially eligible patients (see Fig. 2, Patient inclusion flow chart). Of the 21 eligible patients with septic shock and more than 72 h of intubation, we missed one and two withdrew their consent resulting in 18 included patients. Twelve patients (66.7%) were directly hospitalized in the ICU and the six others (33.3%) were admitted to the ICU after hospitalization in another department.

### Outcomes

Table 1 and supplementary table 1 show the general data concerning this patient population, their length of hospital stay, predicted mortality according to the SAPS II score, organ failure according to the SOFA score and comorbidities, namely COPD, diabetes, cancer and acute kidney injury. The overall length of stay in the ICU was 14.9 ± 9.1 days, with 10 patients (55.5%) staying less than 10 days, 4 (22.2%) between 10 and 24 days and 4 (22.2%) between 24 and 31 days.

**Table 1:** Included population’s general data, SOFA score, SAPS II score and comorbidities. Detailed patient characteristics are available in supplementary table 1 in the electronic supplementary material

Population	*n* = 18		
Age (years)	**63.6** ± 11.6		
Sex	14 M (77.8%); 4 F (22.2%)		
BMI	**28.0** ± 5.2		
Length of stay (days)	**14.9** ± 9.1		
SOFA Score	**12.8** ± 3.1		
SAPS II - Score in points	**41.8** ± 11.1		
SAPS II - Score in % predicted mortality	**31.8** ± 19,5%		
Comorbidities	CIP/CIM	Normal	Died
COPD (*n* = 4)	4 (100%)	-	-
Cancer (*n* = 5)	5 (100%)	-	3 (60%)
Diabetes (*n* = 5)	3 (60%)	2 (40%)	-
AKI KDIGO ≥ 1 (*n* = 14)	13 (92.9%)	1 (7.1%)	3 (21.4%)
AKI KDIGO ≥ 3 (*n* = 9)	8 (88.9%)	1 (1.1%)	2 (2.2%)
Overall	16 (88.9%)	2 (1.1%)	4 (2.2%)

**Fig. 3:**
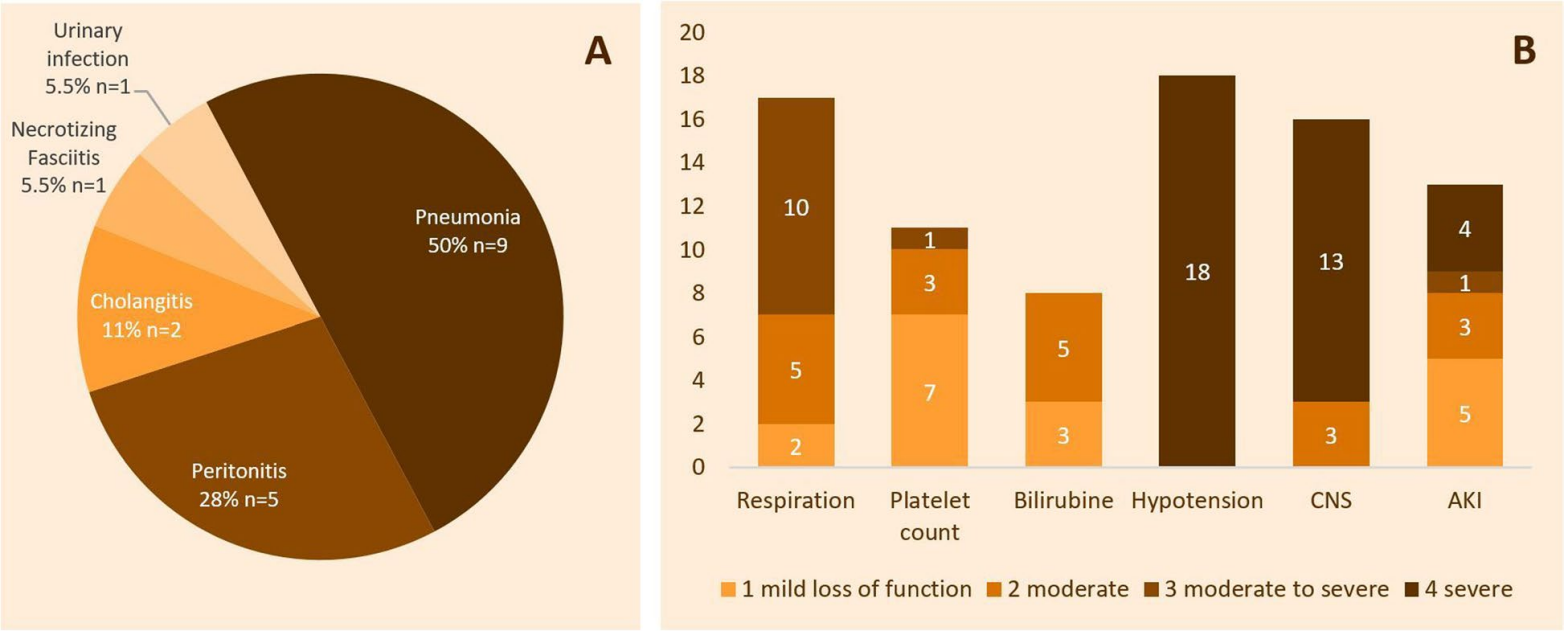
Sepsis etiology and severity on ICU admission. **A** shows the number and percentage of patients per presumed sepsis origin. **B** shows organ failure extent with the number of patients per level of gravity for each item of the SOFA score

At admission, seven patients (38.9%) had continuous noradrenaline administration and seven (38.9%) had a PaO_2_/FiO_2_ ratio inferior to 200. Mechanical ventilation modality was Pressure Support Ventilation (PSV) in thirteen patients (72.2%) and Volume Assisted Control Ventilation (VAC) in the five others (27.7%). The most frequent sepsis etiologies were pneumonia and peritonitis (Fig. 3A). Detailed items of the SOFA score are shown in Fig. 3B and the SOFA scores of each patient are recorded in supplementary table 1.

SOFA score reevaluation in the ICU showed improvement of the septic condition in nine patients (50%), of whom seven showed remission and were transferred from the ICU after 8.6 (± 1.6) days, 1 died and 1 (patient 3) remained hospitalized for 24 days for severe refractory arrhythmia, ventilator associated pneumonia, prolonged weaning and ICU-acquired weakness. The other nine had worsening sepsis and organ failure, six stayed in the ICU for 23.8 (± 8.3) days and three died (See Table 1 and supplementary Table 1).

We recorded failed extubation attempts in five patients (27.8%). Of these, four (22.2%) had prolonged weaning (WIND 3), and one (5.6%) had difficult weaning (WIND 2). Of the whole cohort, five patients (27.8%) had prolonged weaning (WIND 3) taking an average time of 18.8 + 5.9 days, five (27.8%) had difficult weaning (WIND 2), five (27.8%) had short weaning (WIND 1), and three patients (16.7%) died before attempted separation (WIND 0). WIND scores are shown in Table 2.

**Table 2:** Detailed results of nerve conduction study, MRC scale for peripheral muscle weakness and WIND score for diaphragmatic weakness leading to weaning difficulties

N°	First nerve conduction study			**PENT** (mV) evolution R / L (day from intubation)	**Muscle strength** according to **MRC** Pathological below 48/60 points (day from intubation)	**WIND score** 0: died before attempt 1: short 2: difficult 3: prolonged	**Clinical diagnosis**	**Electro-myographical diagnosis**
	**PENT** (mV) R / L (day from intubation)	Sural SNAP (μV) R / L	Sural SNAP velocity in m/s					
**1**	**1.1 / -** **(day 3)**	**7.9 / -**	**56.3**	**- / -**	**-**	**0**	**-**	**CIM**
**2**	**1.3 / -** **(day 3)**	**7.5 / -**	**55.9**	**- / -**	**-**	**1**	**-**	**CIP**
**3**	**0.0 / -** **(day 4)**	**4.0 / -**	**-**	**- / -**	**-**	**1**	Re-admitted for **ICUAW**	**CIP**
**4**	**1.1 / -** **(day 5)**	**13.0 / -**	**44.8**	**0,6 / -** **(day 19)**	**38** **(day 26)**	**3**, can be explained by COPD	**ICUAW** of limbs	**CIM**
**5**	**1.7 / -** **(day 17)**	**7.0 / -**	**50.4**	**1.3 / 1.7** **(day 24)**	**60** **(day 34)**	**3**	**ICUAW** of diaphragm	**Normal**
**6**	**0.8 / 0.3** **(day 9)**	**- / -**	**-**	**- / -**	**-**	**1**	**-**	**Possible CIP/CIM but no SNAP**
**7**	**0.9 / 0.9** **(day 5)**	**0.0 / 0.0**	**-**	**- / -**	**45** **(day 17)**	**3**	**ICUAW** of limbs and diaphragm	**CIP, CIM**
**8**	**0.2 / 0.5** **(day 7)**	**0.0 / -**	**-**	**0.4 / -** **(day 14)**	**-**	**0**	**-**	**CIP**
**9**	**2.2 / -** **(day 4)**	**0.0 / -**	**-**	**1.4 / 0.7** **(day 7)**	**27** **(day 20)**	**3**, can be explained by COPD	**ICUAW** of limbs	**CIP**
**10**	**3.0 / 2.5** **(day 3)**	**- / -**	**-**	**1.4 / 1.2** **(day 7)**	**-**	**2**	**ICUAW** of diaphragm	**Possible CIP/CIM but no SNAP**
**11**	**0.7 / 0.6** **(day 9)**	**- / -**	**-**	**0.6 / 1.7** **(day 16)**	**33** **(day 13)**	**2**, can be explained by COPD	**ICUAW** of limbs	**Possible CIP/CIM but no SNAP**
**12**	**0.5 / 0.6** **(day 4)**	**- / -**	**-**	**- / -**	**59** **(day 4)**	**2**	**ICUAW** of diaphragm	**Possible CIP/CIM but no SNAP**
**13**	**2.1 / 2.4** **(day 7)**	**- / -**	**-**	**- / -**	**-**	**2**	**ICUAW** of diaphragm	**Normal**
**14**	**1.3 / 1.4** **(day 4)**	**3.8 / 0.0**	**61.4**	**- / -**	**-**	**2**	**ICUAW** of diaphragm	**CIP**
**15**	**0.3 / -** **(day 4)**	**0.0 / 1.5**	**-**	**0.1 / 0.4** **(day 8)**	**18** **(day 14)**	**1**	**ICUAW** of limbs	**CIP**
**16**	**0.0** **(day 4)**	**- / -**	**-**	**- / -**	**-**	**0**	**-**	**Possible CIP/CIM but no SNAP**
**17**	**0.7 / 2.6** **(day 7)**	**7.3 / -**	**74.1**	**- / -**	**38** **(day 8)**	**1**	**ICUAW** of limbs	**CIM**
**18**	**1.1 / 1.7** **(day 3)**	**- / -**	**-**	**2.2 / 1.8** **(day 7)**	**-**	**3**, can be explained by COPD	**-**	**Possible CIP/CIM but no SNAP**

### Neurophysiological studies

A first neurophysiological study took place after a median time of four (3.75—7) days after intubation (Absolute range: 3—17), at which time all patients, except one were sedated and none received NMDA blockers. We performed between one and three PENTs (1.67 ± 0.75) for each patient, all of which we considered for assessing occurrence and evolution of nerve impairment.

Seventeen of the eighteen patients (94.4%) had abnormal PENT and 12 (66.7%) were tested further by SNAP (see Table 2). Among these 12, we confirmed CIP, CIM or both in 10 (83.3%) and the two others showed no signs of nerve conduction impairment (16.7%). Specifically, six (33.3%) showed evidence of CIP on CMAPs of median or musculocutaneous nerves and on SNAPs, three (16.7%) showed evidence of CIM with positive CMAP and lengthened conduction time. One patient (5.6%) had evidence of both CIP and CIM and one had unilateral pathological PENT but no amplitude loss in either motor or sensory responses in other parts of the body and was therefore considered a false positive. The last patient showed normal bilateral PENT. The six patients tested only by PENT (33.3% of our population of eighteen) showed bilateral amplitude loss on one (*n* = 4, 22.2%) or two (*n* = 2, 11.1%) tests.

### MRC strength assessment

To validate muscle weakness in early detected CIP and CIM patients, we performed MRC on eight of the 18 patients (44.4%), possible after an average time of 17.0 (± 9.0) days after intubation (Table 2). The eight patients obtained a median score of 38 (31.5 – 48.5) points (Absolute range: 18 – 60, values are shown in Table 2). Six showed insufficient muscle contraction and two normal muscle strength, one of whom had normal ENMG findings and the other bilateral loss of amplitude at PENT.

One of the two patients with normal nerve conduction (patient 5, see Tables 2 and 3) had the longest ICU stay of our cohort (36 days). The patient showed isolated abnormal PENT without loss of amplitude elsewhere on motor upper limb or on sensory stimulation. We also tested the patient by MRC muscle strength test at the end of stay that showed preserved muscle strength. Weaning him from mechanical ventilation was however difficult as 15 days passed between the first separation attempt and successful weaning (WIND score of 3). The patient then recovered with satisfactory muscle contraction and fine mobility 8 days after ICU discharge.

**Table 3:** Ventilation, Sedation and mobilization

N°	**ICU stay length** in days	Mechanical ventilation in days	% of stay with **RASS < -2**	Cumulated doses of **sedatives**			Days from admission to first mobilization				
				**Morphine** equivalent **mg**	**Propofol mg**	**Midazolam mg**	Passive in bed mobilization	Cyclo-ergometer	Out of bed		
									Passive sitting	Active Sitting	Active Upright
1	4	4	**100**	196.15	0	106	3	-	-	-	-
2	8	7	**62.5**	620.90	7426	334	4	-	-	-	-
3	9	7	**77.8**	631.50	878	90	1	-	-	-	-
4	**32**	20	37.5	**1250.92**	15,585	591	6	10	11	12	31
5	**36**	34	**80**	**1792.70**	35,011	564	2	-	34	-	-
6	12	9	**58.3**	539.00	1102	271	4	-	-	10	-
7	**24**	20	**58.3**	**2124.50**	83,987	34	2	18	5	21	22
8	**14**	14	**100**	**1456.60**	24,733	138	7	11	-	-	-
9	**25**	15	44	**1487.70**	13,829	325	4	-	12	-	-
10	9	5	**55.6**	**1286.00**	13,672	116	4	14	-	8	-
11	**17**	8	35.2	702.30	23,343	0	2	4	9	11	16
12	5	5	20	118.00	5721	0	1	3	5	-	-
13	9	7	**66.7**	193.02	5062	157	2	4	-	7	-
14	**19**	11	47.3	**2147.80**	62,050	0	-	-	-	-	-
15	**21**	12	47.6	**1244.60**	12,164	908	5	12	-	12	20
16	5	5	**100**	**1056.90**	3096	66	4	-	-	-	-
17	10	6	40	106.80	2767	99	-	4	7	-	-
18	10	9	**70**	**2249.70**	36,036	79	2	10	-	9	-

### Sedation

Patients were moderately to deeply sedated (RASS < -2) for 59.4% (± 20.9%) of their stay and mechanically ventilated for 77.0% (± 17.7%) of the ICU stay, corresponding to an average time of 11 (± 7.3) days of mechanical ventilation. They received two types of opiates: fentanyl or morphine. Cumulated administered doses of opiates over the whole ICU stay were equivalent to 1067.0 (± 699.2) mg of morphine on average. The average cumulated doses of propofol and benzodiazepines were 19,247.9 (± 22,133.5) mg of propofol and 215.4 (± 241.0) mg of midazolam, respectively. Cumulated doses of sedatives for each patient are shown in Table 3.

### Mobilization rehabilitation

In our ICU, mobilization practices consist of passive in-bed limb exercises during sedation, performed by physiotherapists. Patients can also be mobilized using a cyclo-ergometer or verticalized using the Erigo® robotic bed device. As soon as they are awake, we initiate active mobilization practices of sitting and moving to the upright position. In our cohort, all patients benefited from either passive mobilization during sedation and/or active mobilization after awakening. The different mobilization programs accomplished are shown in Table 3 along with the time from admission to first mobilization. All patients, except one, benefited from passive mobilization at an average time of 77.6 (± 39.1) hours after intubation.

Motorized passive mobilization was performed on ten patients 9 (± 4.8) days after admission. Seven patients (38.9%) were mobilized passively in the sitting position after an average time of 11.9 (± 9.4) days. Eight patients (44.4%) benefited from active mobilization in the sitting position after 11.3 (± 4.1) days after intubation, of whom four (22.2%) also underwent out-of-bed mobilization in the upright position after 22.3 (± 5.5) days.

Six patients (33.3%) neither received sitting nor upright mobilization. Four of them had critical organ failure and died in the ICU. The two others were discharged 8 and 9 days after ICU admission, respectively; one of whom was re-admitted to the unit 16 days after discharge for ICU-acquired polyneuropathy. It took three months from the first ICU discharge for this patient to recover fine motility and muscle strength.

## Discussion

Our patients with septic shock and mechanically ventilated for more than 72 h were moderately to deeply sedated for more than half of their time in the ICU and the average ICU stay length was 14.9 (± 9.1) days. We explored screening for CIP and CIM in this acute phase of sepsis before clinical manifestations of muscle weakness. Indeed, using the simplified screening test proposed by Latronico et al., [13] this is the first study to evaluate septic patients systematically with PENT, around 72 h after intubation. Our results revealed abnormal changes in PENT early during the ICU stay in ventilated patients with septic shock.

The results of this study confirm the likelihood of developing CIP and CIM in patients with MOF, sepsis, and mechanical ventilation [32, 33] and add new information on early nerve conduction disorder in critically ill patients.

The main limitations of this study are the small sample size, the absence of a control group, the collection of all data from a single ICU and the impossibility of obtaining systematic SNAP and MRC testing.

The incidence of CIP and CIM varies in the literature, depending on the studied population. Most of the studies treated in systematic reviews of CIP and CIM concerned patients tested for polyneuropathy after appearance of muscle weakness and absent deep-tendon reflexes [5, 28]. Testing patients in the early phase of ICU stay allowed us to include a more diversified population of septic patients, including those with early sepsis recovery and therefore shorter ICU stay. Of note, even a fast recovery from sepsis does not protect against eventually contracting CIP or CIM from an ICU stay as seen in the three patients hospitalized for less than 10 days, with these conditions revealing themselves after ICU discharge. Indeed, early testing provided evidence that this patient population is at risk of developing CIP or CIM later as seen in the case of one patient who was dismissed from the ICU on day 9 without benefiting from out-of-bed mobilization, and later re-admitted to the ICU for CIP-induced respiratory failure. This patient only recovered muscle strength and fine mobility three months after the first ICU discharge and experienced a very stressful hospitalization.

Systematic acute phase nerve conduction studies on short stay patients offers new diagnostic horizons but is also an organizational challenge, which represents the major limitation of our study. As the study design foresaw a first screening session with PENT and a second for further testing, the second session was not possible for some patients who had either died, left the ICU before further investigation was possible, received muscle relaxants or who were in a state of stress and refused investigation. In addition, the unavailability of nerve conduction studies on some occasions prevented further investigation. Consequently, six patients were only tested with PENT and defined as possible CIP or CIM, without a SNAP analysis allowing differentiating between the CIP and CIM subgroups of ICU-acquired weakness patients. This inconvenience hindered the assessment of possible associations between sedation time and CIP and CIM subgroups. Of these six patients, three showed clinical signs of ICU-acquired weakness of either difficult weaning or limb weakness. Although a reduced CMAP on PENT test was the only electrophysiological finding for these patients, it remains relevant as literature points out an association between isolated reduced CMAP on PENT tests and long-term patient mortality [34, 35].

Essentially, further electrophysiological testing should follow positive PENT screening to allow a more accurate diagnosis of CIP from CIM. Systematic early testing in the ICU is already challenging, and full neurophysiological diagnosis on acute septic patients is remarkably complex in the first acute hospitalization phase. However, as prognosis of CIM is better than CIP [36, 37], systematic positive PENT coupled to SNAPs, muscle conduction time and at least one CMAP on another limb can already be considered an appropriate systematic testing regime [13].

MRC testing of our patients was difficult, as it requires the subject to be awake and cooperative, so we could properly test only those awake and cooperative at the end of their ICU stay with MRC. Of our cohort, MRC was only performed on eight (44.4%) of the 18 patients. Of the 10 other patients, four (22.2%) were never awake to undergo muscle strength testing and died in the ICU. Three (16.7%) did not have a proper state of consciousness to cooperate at ICU discharge. Two (11.1%) were missed because transfer from the ICU occurred so quickly that we had no time to see them before ICU discharge and finally, one (5.6%) was unwilling to be approached on the moment. This again points to the advantages of PENT for early screening, as this method is independent of patient awareness.

Even though MRC muscle strength testing was not systematic, it confirmed the presence of muscle weakness. Six patients presenting loss of amplitude on motor stimulation with PENT also had muscle contraction impairment in the MRC test, corroborating the PENT results. MRC testing also allowed confirming the presence of muscle weakness in one patient not examined by other electrophysiological investigation than PENT (patient 11, see Table 2). One, however, had an important loss of amplitude in PENT but no contraction deficit on the MRC test, raising the question of a possible focal neuropathy of the peroneal nerve (patient 12, see Table 2). In addition, this particular patient had the shortest ICU-stay and time to remission of the whole cohort but still underwent prolonged weaning (WIND 2) with 4 days between the first separation attempt and successful extubation. In the literature, we note that the sole presence of pathological PENT (decreased CMAP) in the early phase of critical illness is associated with an increased 1-year and 5-year mortality [34, 35], even without confirmation by clinically visible limb muscle weakness, indicating that this electrophysiological finding remains a relevant indicator of a patient’s prognosis.

Assessment of the WIND scores helped us to categorize each patient of our cohort for weaning difficulty. With the criteria proposed by Bolton and Latronico for clinical diagnosis of ICU-acquired weakness, all eighteen patients in our cohort (100%) showed either pathological findings on nerve conduction study or clinical signs of ICU-acquired weakness. Clinical signs included an MRC score below 48 testifying limb weakness or a WIND score of 2 or 3 not explained by an underlying lung or heart condition attesting to weakness of the diaphragm (see Table 2). Concerning the two patients with normal nerve conduction tests, as they also presented weaning difficulty, the diagnosis of disuse myopathy caused by prolonged exposure to mechanical ventilation, as described in the Latronico and Bolton review [28], could apply to them as this common entity shows clinical signs of muscle wasting only with no electrophysiological findings.

Early mobilization techniques have been proven beneficial in shortening ICU stay and improving rehabilitation [38]. Specific treatment of CIP and CIM include passive and active mobilization of limb and respiratory muscle through physiotherapy, but these techniques are still lacking in current clinical practice [6, 39]. Beginning mobilization in the first days after onset of critical illness and intubation was reported to be safe and offer a real improvement in rehabilitation and in shortening the length of hospital stay in critically ill patients [38, 40, 41]. Coupled with efficient sepsis treatment [42], early mobilization offers better chances of recovery from many complications associated with critical illness [43]. Indeed, early mobilization, especially verticalization associated with cyclic movement, maintains the neuro-vegetative system by stimulating the endogenous adrenergic system and preventing complications from immobilization such as CIP and CIM [44].

Our study emphasizes the need for early screening of CIP/CIM for three reasons. First, although severe sepsis with MOF and lengthened hospitalization are risk factors, it seems impossible to predict whether a patient is more likely to develop these conditions as we found one patient with severe sepsis and the longest stay who did not show evidence of polyneuropathy. Second, the average state of consciousness of these patients and average time to awakening makes it impossible to diagnose the CIP/CIM by following symptoms. Early neurophysiological screening is therefore necessary to allow early recognition of the disease and to avoid misdiagnosis of a disorder of consciousness and absence of motor response [45]. Third, we can expect benefit from early specific passive muscle stimulation since active mobilization techniques are impossible on sedated patients. Early diagnosis would enable a tailored therapy for these at-risk patients. However, specific benefits need to be evaluated in a larger study with the same inclusion criteria and a protocol focusing on the mobilization procedure.

## Conclusion

CIP, CIM and other peripheral deficits are detectable early by electrophysiological screening with motor and sensory testing in 55.6 to 88.8% of patients with septic shock undergoing mechanical ventilation. Association of CIP/CIM with cancer, acute kidney injury and diabetes is possible and we should explore this in future research. The expected benefit from early screening of CIP and CIM is to allow early active mobilization by physiotherapists if the patient is awake or passive mobilization if still sedated to improve patient outcome. The study opens perspectives for use of emerging mobilization techniques on this category of patients.

This study brings new knowledge on CIP and CIM in ICU patients with mechanical ventilation and septic shock by having systematically screened for them at an early stage of the disease and before symptoms are apparent. It furnishes information concerning early weaned and short stay patients that have not been explored in previous studies, which did not test CIP and CIM systematically.

## Supplementary Information

**Additional file 1: Supplementary Table 1.** Population general data, comorbidities, sepsis and outcome in detail for each patient.

## Data Availability

Most of the data that support the findings of this study are included in the article for publication and its supplementary information file. Restrictions apply to availability of additional data such as patient screening, which requires authorization from the Lausanne ICU for sensitive data. Other data are available from the authors upon reasonable request and with permission from PD-MER Lise Piquilloud Imboden.

## Nerve conduction study

CIP: Critical Illness Polyneuropathy
CIM: Critical Illness Myopathy
PENT: Peroneal Nerve Test
SNAP: Sensory Nerve Action potential

## Scores and scales

SAPS II: Simplified Acute Physiology Score II
SOFA: Sequential Organ Failure Assessment
KDIGO: Kidney Disease Improving Global Outcome
RASS: Richmond Agitation-Sedation Scale
MRC: Medical Research Council
