# Supplementary material for: Early detection of ICU-acquired weakness in septic shock patients ventilated longer than 72 h
Source: BMC Pulm Med. 2022 Dec 6;22:466. doi: 10.1186/s12890-022-02193-7 (PMC9724444; doi:10.1186/s12890-022-02193-7)
Supplement: Supplementary file 1 — Additional file 1: Supplementary Table 1. Population general data, comorbidities, sepsis and outcome in detail for each patient. [file 12890_2022_2193_MOESM1_ESM.docx]

**Early detection of ICU-acquired weakness in septic shock patients ventilated longer than 72 hours**

*Caroline Attwell, Acute Neuro-Rehabilitation Unit, Lausanne University Hospital, Lausanne, Switzerland*

*Laurent Sauterel, Lausanne University Hospital, Lausanne, Switzerland*

*Jane Jöhr, Acute Neuro-Rehabilitation Unit, Lausanne University Hospital, Lausanne, Switzerland*

*Lise Piquilloud, Adult Intensive Care Unit, Lausanne University Hospital and University of Lausanne, Lausanne, Switzerland*

*Thierry Kuntzer, Nerve-Muscle Unit, Neurology Service, Lausanne University Hospital, Lausanne, Switzerland*

*Karin Diserens, Acute Neuro-Rehabilitation Unit, Lausanne University Hospital, Lausanne, Switzerland*

| N° | Age | Sex | Outcome | at Admission | | | |  | Sepsis evolution at day 3 | Dialysis |
| --- | --- | --- | --- | --- | --- | --- | --- | --- | --- | --- |
|  |  |  |  | Comorbidities | **SAPS II - points** | **SAPS II - % Predicted mortality** | **SOFA** | AKI  (KDIGO stage) |  |  |
| 1 | 69 | F | Died | None | **60** | **68,09** | **12** | 0 | Improved | No |
| 2 | 51 | F | Survived | **Cancer** | 39 | 22,96 | **11** | 0 | Improved | No |
| 3 | 76 | M | Survived | **Diabetes** | 47 | 39,19 | **15** | 0 | Improved | No |
| 4 | 61 | F | Survived | **COPD** | 46 | 36,96 | **12** | 0 | **Worsened** | **Yes** |
| 5 | 59 | M | Survived | **Diabetes** | **59** | **66,1** | **14** | **3** | **Worsened** | **Yes** |
| 6 | 62 | M | Survived | **Diabetes** | 34 | 15,29 | **16** | 0 | **Worsened** | **Yes** |
| 7 | 79 | M | Survived | **Cancer** | 23 | 5,22 | **10** | 0 | Improved | No |
| 8 | 73 | M | Died | **Cancer** | 45 | 34,77 | **11** | 0 | **Worsened** | No |
| 9 | 70 | M | Survived | **COPD** | **57** | **61,93** | **15** | **3** | **Worsened** | **Yes** |
| 10 | 31 | M | Survived | None | 43 | 30,56 | **16** | **3** | Improved | **Yes** |
| 11 | 61 | M | Survived | **COPD** | 51 | 48,39 | **15** | **3** | **Worsened** | **Yes** |
| 12 | 49 | M | Survived | None | 22 | 4,67 | 9 | **1** | Improved | No |
| 13 | 76 | F | Survived | **Diabetes** | 43 | 30,56 | **10** | 0 | Improved | No |
| 14 | 74 | M | Died | **Cancer** | 34 | 15,29 | 7 | 0 | **Worsened** | **Yes** |
| 15 | 58 | M | Survived | **Diabetes** | 48 | 41,46 | **18** | **3** | **Worsened** | **Yes** |
| 16 | 72 | M | Died | **Cancer** | 29 | 9,67 | **18** | **3** | **Worsened** | **Yes** |
| 17 | 68 | M | Survived | None | 43 | 30,56 | **13** | **3** | Improved | No |
| 18 | 56 | M | Survived | **COPD** | 30 | 10,64 | 9 | **1** | Improved | No |

**Supplementary table 1:** Population general data, comorbidities, sepsis and outcome in detail for each patient
